# Pulmonary delivery of insulin by dry powder inhaler formulations

**DOI:** 10.22038/ijbms.2025.83954.18168

**Published:** 2025

**Authors:** Mohsen Imenshahidi, Mona Kabiri, Mohammad Jandaghi, Seyed Salman Razavi Rouhani, Khalil Abnous, Gholamreza Karimi, Mohsen Tafaghodi

**Affiliations:** 1 Pharmaceutical Sciences Research Center, Mashhad University of Medical Sciences, Mashhad, Iran; 2 School of Pharmacy, Mashhad University of Medical Sciences, Mashhad, Iran; 3 Nanotechnology Research Center, Pharmaceutical Technology Institute, Mashhad University of Medical Sciences, Mashhad, Iran; 4 Student Research Committee, Mashhad University of Medical Sciences, Mashhad, Iran

**Keywords:** Diabetic rats, Dry powder inhalers (DPIs), Glucose, Insulin, Pulmonary administration

## Abstract

**Objective(s):**

Insulin therapy is critical in diabetic patients for controlling blood glucose levels. In recent years, pulmonary insulin delivery has emerged as an alternative approach for overcoming the therapeutic disadvantages of subcutaneous insulin administration, such as pain, infection risk, and needle phobia. To develop the pulmonary insulin formulation, five insulin-containing dry powder inhalers (DPIs) with different excipients were tested in diabetic rats.

**Materials and Methods:**

Formulations were inoculated endotracheally to diabetic rats induced by streptozotocin. Insulin and glucose assays were performed on blood samples taken from the carotid artery at different intervals, including baseline and 1–4 hr after insulin administration.

**Results:**

The results illustrated that five formulations (F1-F5) could gradually increase the plasma insulin level during time points of the study. The first and third formulations comprising insulin, mannitol, and sodium citrate in the absence (F1) or presence of sodium alginate (F3) also declined plasma glucose levels in animals.

**Conclusion:**

The results confirmed that the pulmonary formulations could deliver and release insulin molecules in a good manner, and the biological activity of the two formulations, including F1 and F3, is acceptable and comparable to the subcutaneous insulin. Our findings support that the mentioned DPI products could have therapeutic potential as an alternative to subcutaneous insulin. Further investigations are needed to prove the capability of F1 and F3 spray-dried products to treat diabetic individuals.

## Introduction

In 1921, Banting and Best discovered insulin as a hypoglycemic agent, and various routes of administration, such as subcutaneous (SC), intramuscular (IM), intravenous (IV), oral, pulmonary, nasal, sublingual, etc., were explored in the years following (1, 2). Although many delivery systems can potentially control hyperglycemia, the SC injection is the standard and main route for insulin administration (1). Subcutaneous administration, especially in long-term use of insulin, has several problems, such as needle phobia, injection pain, lipodystrophy, peripheral hyperinsulinemia, and noncompliance (3). 

Various dosage forms and delivery systems have been tested for the non-invasive routes of insulin to resolve the mentioned problems, and pulmonary administration has shown high therapeutic value for the delivery of insulin (4, 5). The pulmonary route can bypass the “first pass metabolism” (3) and has extensive vasculature and permeable epithelium (6). The FDA approved the first pulmonary formulation of insulin in 2006 (Exubera^®^, Pfizer company) (4). However, patients and physicians did not prefer it to the routine parenteral formulation, and the manufacturer withdrew the mentioned product due to the high cost, the bulky device, and concerns regarding its function (3). In June 2014, the second pulmonary insulin was launched in the United States (Afrezza^®^, Sanofi company) for patients with type 1 diabetes mellitus (T1DM) (3). This formulation was approved as a prandial insulin and has been based on Technosphere^®^ dry powdered inhaler (DPI) formulation. As an ideal postmeal glucose control, the hypoglycemic effect of Afrezza^®^ starts after 15 min and lasts 2 to 3 hours (7). The initial side effects of Afrezza^®^ are transient cough and a reduction in lung function (8). Other pulmonary delivery devices, such as the AERx^®^ insulin management system, AIR^®^, and Dance 501^®^, were evaluated in clinical trials (3). The AERx^®^ inhaler device containing insulin liquid aerosols and AIR^®^ as the mechanical system with insulin dry powder completed phase III clinical trials. However, the manufacturers discontinued further investigations to attain FDA approval (9). The vibrating mesh nebulizer of insulin, Dance 501^®^, passed all clinical trial phases and was approved by the FDA. Due to the low flow rate of the mentioned system, patients needed to take more breaths to complete the inhaled dosage, which became the less favorable device for diabetic individuals (10). Nevertheless, there is abundant attention to pulmonary insulin delivery (4) due to the therapeutic potential of inhaled formulations.

In our previous study, insulin powders with different excipients were prepared using the spray drying method (11). The fabricated DPIs revealed a desirable aerodynamic particle size for intrapulmonary delivery (1–5 µm). Various excipients, including mannitol, sodium citrate, L-alanine, sodium alginate, chitosan, or DPPC, have been utilized to obtain physical and chemical stability of insulin and improve aerosolization performance to enhance pulmonary absorption. Mannitol and sodium citrate were applied as the primary excipients in all DPI formulations, and other components were used to reduce mannitol crystallization during spry-drying and increase insulin pulmonary delivery. Among them, insulin formulations containing mannitol, sodium citrate, and alginate were defined as the potential products for pulmonary delivery due to several advantages, including high yield with ideal aerosolization properties, appropriate insulin release, and amorphous shape (11). 

Various excipients, such as mannitol, sodium citrate, and amino acids, have been efficient in inhalation delivery to produce stable spray-dried insulin products at room temperature (12). Mannitol and sodium citrate were the two excipients utilized in DPI human insulin formulation (Exubera^®^, Pfizer company) (13, 14). Mannitol is generally recognized as safe (GRAS) and used as the hydrophilic particulate matter in spray-dried inhalation formulations (15). Mannitol can be applied as a stabilizer in the presence of other excipients due to mannitol’s low glass transition temperature (Tg, 11 °C), which tends to re-crystallize and denature insulin in the spray drying process (16). The mentioned excipient at low humidity and amorphous shape can preserve the stability of proteins via hydrogen interactions in pulmonary formulations (16). Some studies reported that utilizing small molecules such as sodium citrate, sodium phosphate, and amino acids can modify the solid-state properties of mannitol carriers (12).

Using amino acids such as alanine, leucine, and glycine in inhalation products revealed decreasing the hygroscopicity of particles, increasing the aerodynamic property of powders and aerosol stability as well as protein protection against denaturation and thermal procedures (13, 16). The pulmonary toxicity and systemic absorption of amino acids are unknown. However, amino acids as endogenous excipients have no critical risk of lung toxicity (13). 

Alginate is a natural polymer that performs as an alternative excipient for pulmonary delivery to the lung due to its desirable aerosolization performance to produce inhalable particles (17, 18). The proper aerodynamic distribution of particle size and controlled-release drug or protein profiles have been reported for pulmonary formulations with alginate, demonstrating a high potential of the mentioned biopolymer in inhalation delivery. Alginate is a non-toxic and biocompatible polysaccharide, and sustained insulin release for more than six hours was reported for DPI formulations in the presence of alginate (18). 

Chitosan is a non-toxic and non-irritant polycationic polymer with mucoadhesive absorption enhancer properties. It improves the retention time of inhalation products due to interaction with mannose receptors expressed on alveolar macrophages (13). Accordingly, this biodegradable and biocompatible polysaccharide is a desirable carrier for the controlled-release delivery of inhalation particles into the lungs (15). The inhalation administration of insulin encapsulated into trimethyl chitosan considerably increased insulin absorption and enhanced the bioavailability of spray-dried microparticles (19). Chitosan has not yet received FDA approval for pharmaceutical usage due to concerns regarding its purity, sources, and inflammatory responses, and it needs regulatory approval before utilization (20).

Lipids such as DPPC and cholesterol are promising alternatives as endogenous excipients to the lungs with confirmed safety profiles. These are quickly metabolized and cleared via absorption by epithelial cells or macrophage uptake (13). DPPC is known as a natural phospholipid that is present in the lung surfactant system. DPPC is generally recognized as safe and acts as a pulmonary absorption enhancer to increase the efficient penetration of particles in DPI formulations. Moreover, this phospholipid can reduce the surface energy of particles and the cohesive property of spray-dried powder and facilitate particle formation during the atomization process (13, 15). The AIR^®^ insulin dry powder inhalation product containing DPPC and sodium citrate excipients had safety and efficiency with suitable aerosolization performance and passed the phase III clinical trial. No adverse effects regarding the DPPC component used in AIR^®^ formulation were reported at the cellular level (21). 

In the present investigation, the potential of five insulin formulations containing dry powder inhalers with different excipients has been evaluated to promote pulmonary absorption of insulin and bioavailability in diabetic rats induced by streptozotocin (STZ). 

## Materials and Methods

### Insulin-containing DPI formulations

Streptozotocin, Heparin, and Urethane were purchased from Sigma (USA). Sodium citrate was obtained from Merck (Germany). Chitosan (low molecular weight with low viscosity) and insulin were obtained from Fluka (Japan) and Novo Nordisk (Denmark), respectively. As described in our previous study, the insulin-containing DPI formulations (F1-F5) with different excipients were prepared using the spray-drying technique (11). All pulmonary formulations containing insulin, mannitol, and sodium citrate in the absence (F1) or presence of L-alanine (F2), sodium alginate (F3), chitosan (F4), or L-alanine and dipalmitoylphosphatidylcholine (DPPC; F5). 

### In vivo study

*Induction of experimental diabetes*

The present study was accomplished on Wistar rats (male, 250–300 g) from Razi Institute, Mashhad, Iran. Animal experiments were applied based on the guidelines provided by the Mashhad University of Medical Sciences ethics committee.

Animals were made diabetic via intraperitoneal injection of streptozotocin (45 mg/kg). Citrate buffer was injected into the control animals (22). One week later, the diabetes was approved using blood sampling from the tail vein, and the glucose levels were detected with a glucometer (Accu-Chek, Germany). Blood glucose concentrations of more than 300 mg/dl were considered a limit for diabetic rats (23). STZ-induced diabetic rats fasted for 12 hr before administration of insulin formulations, and animals had free access to water.

*Endotracheal administration of insulin*

The method of Brown *et al*. (24) was applied for endotracheal administration of formulations. Animals were secured on their backs, adjusted to 45°-60° angles, and anesthetized using an intra-peritoneal urethane administration (1.5 g/kg). Anesthesia was checked with footpad reflex, and insulin formulations (F1-F5) were insufflated to rats. The positive control group received insulin subcutaneously. The animals had spontaneous breathing during the study. The details of treatment groups and administration routes are summarized in Table 1.

An outer light source was concentrated on the animal trachea for better illumination of the trachea. Each powder dose was poured into a powder insufflator device (Penn-century, USA) with a 12 cm delivery tube. The device was inserted into the rat trachea, and powder (containing 2 IU of insulin) was gradually delivered by an air-filled syringe connected to the device. After administration, blood samples were taken and collected into EDTA tubes immediately before insulin dosing and at intervals of 1–4 hr after dosing to assay the glucose and insulin. Rats were randomly divided into six groups of four rats, and five groups received pulmonary formulations of insulin-loaded particles (F1-F5). As the control, one group received a subcutaneous injection of 2 IU insulin into the dorsal flank. 

*Blood sampling*

Blood samples were collected from the carotid artery. The left carotid artery was cannulated using a heparinized saline cannula (50 U/ml). Subsequently, samples were collected from the carotid artery at times 0, 1, 2, 3, and 4 hr. The serum was separated by centrifugation (4 min at 3,000 g and 4 °C) and kept at -20 °C until insulin and glucose analysis. 

*Serum insulin and glucose measurements*

Insulin concentrations were detected using an enzyme-linked immunosorbent assay (ELISA) based on the manufacturer’s protocol (Monobind Inc, CA, USA). The serum glucose levels were determined by a glucometer (Accu-Chek, Roche, Germany). The levels of insulin (µIU/ml) and glucose (mg/dl) are reported as mean±SEM (*n*=4) for each treatment. 

### Statistical analysis

Data were analyzed using GraphPad Prism version 5.0 software. All formulations were compared using two-way ANOVA followed by Tukey’s multiple comparison test. *P*-values<0.05 were defined as significant.

## Results

**Figure 1:**
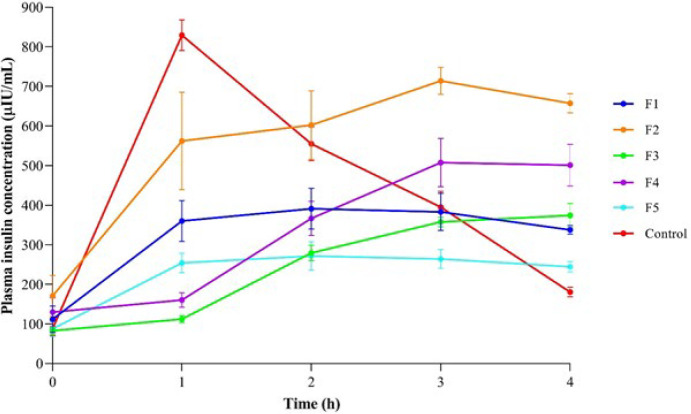
Plasma insulin concentrations following administration of DPI formulations (F1–F5) or subcutaneous insulin to STZ-diabetic rats during the intervals of the study

In the current study, several excipients have been tested to compare their efficiency in improving pulmonary insulin delivery in a diabetic animal model. Figure 1 compares insulin plasma levels after administering spray-dried formulations (F1-F5) and subcutaneous insulin. Drug release from pulmonary formulations was assessed up to 4 hr, and five designed products revealed a plateau state before the fourth hour. All DPI formulations gradually increased insulin levels during the time points of the study, while the SC insulin injection showed a burst release within the first hour (Figure 1). 

DPI formulations in the absence (F1, *P*=0.003) or presence of sodium alginate (F3, *P*=0.001), chitosan (F4, *P*<0.001), or L-alanine and DPPC (F5, *P*<0.001) significantly released lower insulin levels than the control at the first hour after administration, with no significant difference between F2 and the control group (*P*=0.488). In the second hour, no significant differences were found regarding insulin levels between the subcutaneous insulin and spray-dried F1 (*P*=0.276), F2 (*P*=0.993), or F4 (*P*=0.130) products (Table 2). Moreover, the spray-dried product containing L-alanine (F2) could significantly raise insulin concentrations in the third (*P*=0.012) and fourth (*P*=0.001) hour after injection compared to the control group, whereas F1 (*P*=0.001) and F3 (*P*=0.035) considerably released the drug at the end time point of this study. No significant differences were found between the five DPI formulations and subcutaneous insulin delivery at the baseline (Table 2).

**Figure 2:**
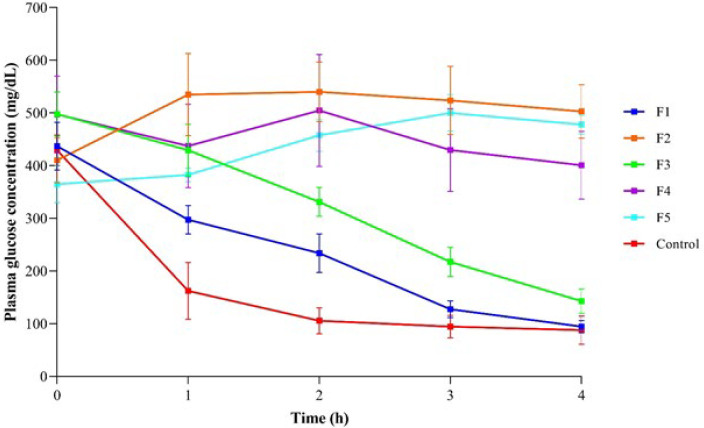
Assessment of plasma glucose levels in STZ-diabetic rats followed by pulmonary formulations (F1-F5) or subcutaneous insulin administration within different time points

As shown in Figure 2, the glucose levels have been assessed between the subcutaneously administered insulin and pulmonary products. Among formulations, F1 and F3 could reduce glucose throughout four hours in diabetic rats, whereas other products increased plasma glucose compared to the control group. 

Table 2 demonstrates no significant differences regarding plasma glucose levels between F1 or F4 products and SC insulin injection during the intervals assessed in our study. On the other hand, F2 and F5 significantly enhanced glucose levels within 2 to 4 hours after post-treatment. Although F3 could significantly elevate plasma glucose levels relative to the control group two hours following administration (*P*=0.006), there were no significant differences at other post-treatment intervals.

**Figure 3:**
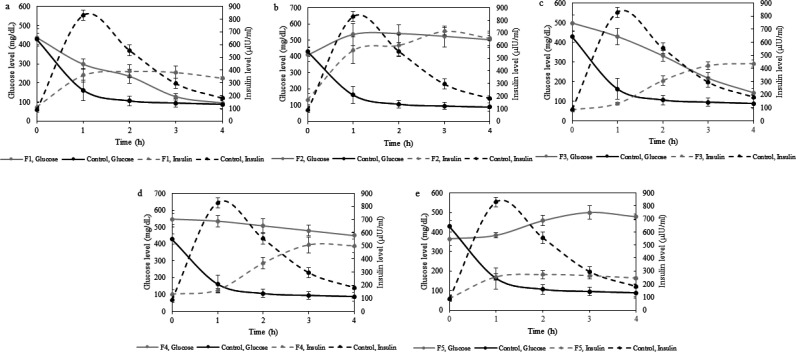
Plasma insulin and glucose levels following spray-dried formulations (F1-F5) or subcutaneous insulin administration to STZ-diabetic rats

Figure 3 synchronously illustrates the changes in plasma insulin and glucose levels of formulations. According to the results, F1 and F3 illustrated a good relationship between insulin release and plasma glucose levels (Figure 3a and 3c). The mentioned products were determined as the potential spray-dried formulations in the present study.

## Discussion

In this investigation, the therapeutic potential of the insulin-containing DPI formulations with various excipients was assessed in diabetic rats induced by streptozotocin. The results indicated that all formulations (F1-F5) could moderately release drugs and increase plasma insulin levels. Due to the controlled drug release by pulmonary formulations, the insulin levels gradually elevated throughout four hours compared to the SC insulin injection and reached the plateau. In addition, spray-dried products comprising insulin, mannitol, and sodium citrate in the absence (F1) or the presence of sodium alginate (F3) also decreased plasma glucose levels during four hours in rats.

The present study’s findings are compatible with our previous *in vitro* evaluation of the mentioned five pulmonary formulations (11), and the profiles of insulin release in the two studies are the same for mentioned products. 

Compared to subcutaneous insulin injection, F1 demonstrated a more uniform and steady insulin release profile during four hours of study. Therefore, it seems that D-mannitol, as the main excipient of F1, had an appropriate property for releasing insulin molecules. The effect of F1 on plasma glucose level was consistent with its insulin release profile and subcutaneous insulin. Mannitol represents a potential glycemic control agent with an antihyperglycemic activity, which suppresses the absorption of small intestinal glucose and elevates glucose uptake of muscles to control postprandial blood glucose enhancement (25). 

Skyler *et al*. reported similar results regarding the mentioned excipient in pulmonary and subcutaneous human insulin delivery in diabetic patients (26). Additionally, mannitol administration induced glycemic control in diabetic rats and inhibited the activity of α-glucosidase and α-amylase by impairing the digestion of carbohydrates (25). This study revealed that mannitol significantly suppressed the absorption and diffusion of glucose in healthy and diabetic groups. The potential of mannitol for controlling glycemia by impairing carbohydrate digestion was suggested in previous studies. Mannitol can suppress the increase of postprandial blood glucose and improve glucose uptake in diabetic rat models (25).

F3 also illustrated a proper profile of the insulin release during four hours. The glucose-lowering effect of the mentioned formulation is comparable to subcutaneous control after two hours. Mannitol and alginate compose the main excipients of the mentioned DPI product. Alginate, as the non-toxic and non-immunogenic excipient, has desirable mucoadhesive and gel-forming properties (27) as well as biological and minor glucose-lowering activities (28). Moreover, the appropriate aerodynamic attributes and particle shape of F3 could be determinative in glucose-lowering and insulin release.

Alginate, a hydrophilic polysaccharide, has a molecular mass from 12 to 180 kDa, and a polymer with a molecular weight between 50 and 100 kDa can inhibit the enhancement of insulin and glucose levels. Alginate can interact with cationic metals such as Ca2+, and due to the deprotonation of carboxyl groups at acidic pH, the cross-linking of ions arises to form a polymer matrix utilized in drug or insulin formulations (29). Sodium alginate can temporarily open the tight junction of epithelial cells and increase the permeability of insulin molecules (30).

Chai *et al*. suggested that subcutaneous insulin delivery using alginate in the absence or presence of glucose oxidase had efficient hypoglycemic effects with acceptable glucose tolerance relative to the free insulin in diabetic rats (31). The glucose tolerance induced by sodium alginate may be caused by suppressing glucose absorption from the small intestine via the gelation of the free alginic acid in the stomach (32). Husni *et al*. also described that the oral administration of sodium-alginate considerably reduced pre and postprandial glucose levels in diabetic rats (33). Their findings revealed greater glycemic effectiveness of high alginate dosages than the low doses. Based on previous studies, the alginate-based insulin delivery systems improve the efficiency of insulin, hypoglycemic drugs, and phytochemical medicines, thereby augmenting the therapeutic effectiveness against DM (34).

Although F2, F4, and F5 could increase plasma insulin levels during the different post-treatment time points, no suitable findings regarding plasma glucose levels were observed in our study. This discrepancy between insulin release and glucose activity can be explained based on different excipient usage in the mentioned formulations. 

The presence of alanine in F2 and F5 enhanced glucose plasma levels, and some studies demonstrated this biological activity of the alanine component (35-38). L-alanine increases pancreatic β-cell glucose metabolism, inducing insulin secretion via cotransport with Na^+^. Co-transportation leads to the opening of L-type calcium channels, depolarization of the β-cell membrane in the presence of glucose, and evoking calcium influx (39, 40). Genuth *et al*. reported that the oral administration of alanine significantly improved plasma glucose levels in insulin-dependent diabetic individuals (41). Other researchers also revealed that intravenous injection of alanine solution in healthy and obese participants considerably elevated insulin and glucose levels (42). The lack of post-meal glucose concentration suppression may be due to the consumption of the alanine carbon chain in gluconeogenesis, which proceeds via the liver (39).

In addition to the alanine excipient, the spray-dried F5 product contains the DPPC, resulting in a high glucose level in diabetic rats within four hours. Although phospholipids improve the permeability of molecules within membranes as absorption enhancers, they can damage epithelial cell membranes (43, 44).

 However, Chono *et al*. reported that the liposomal form of insulin in the presence of DPPC could increase the pulmonary delivery of insulin due to opening the tight junction of alveolar epithelial cells without mucosa damage (45, 46). Their findings revealed that the presence of the palmitoyl group in phospholipids such as DPPC resulted in liposomes’ improvement in insulin absorption. On the other hand, the insulin loaded in DPPC liposomes had higher pulmonary absorption effectiveness compared to the co-incorporation of insulin and DPPC liposomes or free insulin (46). The mentioned enhancing effect was also decreased for the aerosolized DPPC liposomes with a particle size of 1 µm relative to the 100 nm particles due to the enhancement of uptake via alveolar macrophages (45). The authors suggested that the phospholipid type and particle size are two essential factors for pulmonary insulin transport. 

A study evaluated the incorporation of liposomes and DPPC in improving insulin pulmonary delivery in type II cells (47). The DPPC alone demonstrated high glucose levels in nasal lavage fluids and saline buffer, and no hypoglycemic effects were detected. The physical mixture of insulin with DPPC or liposomes could reduce blood glucose in nasal lavages and saline buffer for four hours, with a significant difference between the mentioned groups. The insulin molecules can interact with the phospholipid surfaces via hydrophobic binding, and the binding degree is related to the hydrocarbon chain structure (47, 48).

The review study conducted by Weijers described that saturated phospholipids such as DPPC could reduce the capacity for glucose transport across cell membranes due to increasing the van der Waals interactions between hydrocarbons and decreasing the flexibility of the plasma membrane (49), which is consistent with our findings.

Previous studies proved that chitosan has blood-glucose-lowering and antidiabetic properties with no adverse effects. As a bioactive component with biological activity, chitosan induces the PI3K/AKT pathway to stimulate insulin signaling. Additionally, the antihyperglycemic effect of chitosan is related to increased glucose metabolism and decreased pancreatic β-cells dysfunction by suppression of α-glucosidase and α-amylase activities. Consequently, chitosan can enhance insulin secretion, improve gut microbiota, and reduce insulin resistance (50, 51).

The pulmonary F4 contains chitosan with low molecular weight (MV) and viscosity, revealing a high level of plasma glucose two hours following administration. According to previous investigations, low MW chitosan (LCH) had a hypoglycemic effect on diabetic animals (52, 53), while some of them indicated the role of high MV chitosan (HCH) in decreasing glucose concentration (54, 55). Yao *et al*. demonstrated that HCH was more efficient in decreasing plasma glucose levels compared to LCH in STZ-diabetic rats (54). Moreover, another study suggested that chitosan with high MV significantly reduced glucose levels in diabetic rats relative to the LCH. Although the LCH had efficacy in reducing the plasma insulin concentration, the glucose level was enhanced in the group that received LCH relative to diabetic rats and control groups (55). 

**Table 1:** Details of vaccine groups are presented, including the number of inoculated mice, the type of treatment, and the administration routes

Groups	Number of rats	Treatment	Administration
Control	4	Insulin	Subcutaneous
F1	4	Insulin+Mannitol+Sodium citrate	Pulmonary
F2	4	Insulin+L-Alanine+Mannitol+Sodium citrate	Pulmonary
F3	4	Insulin+Sodium alginate+Mannitol+Sodium citrate	Pulmonary
F4	4	Insulin+Chitosan+Mannitol+Sodium citrate	Pulmonary
F5	4	Insulin+L-Alanine+DPPC+Mannitol+Sodium citrate	Pulmonary

Five pulmonary formulations compose insulin, mannitol, and sodium citrate in the absence (F1) or presence of L-alanine (F2), sodium alginate (F3), chitosan (F4), or L-alanine and DPPC (F5).

Values are presented as the mean±SEM of four experiments. Five pulmonary formulations compose insulin, mannitol, and sodium citrate in the absence (F1) or presence of L-alanine (F2), sodium alginate (F3), chitosan (F4), or L-alanine and DPPC (F5). STZ: Streptozotocin; DPI: Dry powder inhaler

**Table 2:** *P*-value of various DPI formulations (F1-F5) versus the control group (subcutaneous insulin) regarding plasma insulin and glucose levels at different study time points

Time (hr)	F1		F2		F3		F4		F5	
	Insulin	Glucose	Insulin	Glucose	Insulin	Glucose	Insulin	Glucose	Insulin	Glucose
Pre, 0	0.984	> 0.999	0.679	0.998	> 0.999	0.752	0.888	0.935	0.718	> 0.999
Post, 1	0.003*	0.368	0.488	0.097	0.001*	0.089	< 0.001*	0.227	< 0.001*	0.110
Post, 2	0.276	0.177	0.993	0.011*	0.021*	0.006*	0.130	0.1379	0.025*	0.004*
Post, 3	> 0.999	0.803	0.012*	0.023*	0.927	0.091	0.659	0.096	0.203	0.001*
Post, 4	0.001*	> 0.999	0.001*	0.007*	0.056	0.649	0.035*	0.059	0.101	< 0.001*

* Significant *P*-value. Five pulmonary formulations compose insulin, mannitol, and sodium citrate in the absence (F1) or presence of L-alanine (F2), sodium alginate (F3), chitosan (F4), or L-alanine and DPPC (F5). DPI: Dry powder inhaler

Data are expressed as the mean±SEM of four experiments. Five spry-dried formulations compose insulin, mannitol, and sodium citrate in the absence (F1) or presence of L-alanine (F2), sodium alginate (F3), chitosan (F4), or L-alanine and DPPC (F5). STZ: Streptozotocin

Data are represented as the mean±SEM of four experiments. Five spry-dried formulations compose insulin, mannitol, and sodium citrate in the absence (F1) or presence of

L-alanine (F2), sodium alginate (F3), chitosan (F4), or L-alanine and DPPC (F5). STZ: Streptozotocin

## Conclusion

The findings of the present *in vivo* study confirmed that five pulmonary formulations could deliver and gradually release insulin molecules in a good manner. Moreover, two DPI formulations, including F1 and F3, can be considered alternatives to subcutaneous insulin due to their potential to decrease plasma glucose concentrations in diabetic rats. Alanine and alginate are the best excipients for pulmonary insulin delivery among the excipients examined in this study. Further studies are suggested to approve the therapeutic potentials of the two mentioned DPI products. 

## Declaration

We have not used any AI tools or technologies to prepare this manuscript.
